# *N*-(2-Oxo-2,3,4,5,6,7-hexa­hydro-1*H*-azepin-3-yl)cyclo­hexa­necarboxamide

**DOI:** 10.1107/S1600536813031863

**Published:** 2013-11-30

**Authors:** Shi Chunjuan, Yang Zhao

**Affiliations:** a Suzhou University Experimental Material Supply Center, Suzhou 215123, People’s Republic of China; b College of Pharmacy, China Pharmaceutical University, Tongjiaxiang No. 24 Nanjing, Nanjing 210009, People’s Republic of China

## Abstract

In the title compound, C_13_H_22_N_2_O_2_, both the six-membered ring and the seven-membered lactam ring adopt chair conformations. In the crystal, mol­ecules are linked by pairs of N—H⋯O hydrogen bonds between inversion-related lactam rings into centrosymmetric dimers with an *R*_2_^2^(8) graph-set motif. Further N—H⋯O hydrogen bonds link the molecules into [100] chains.

## Related literature

For background information on 3-(acyl­amino)­azepan-2-ones, see: Fox *et al.* (2009 ▶); Grainger & Fox (2006 ▶). For a related crystal structure, see: Zhu *et al.* (2007 ▶).
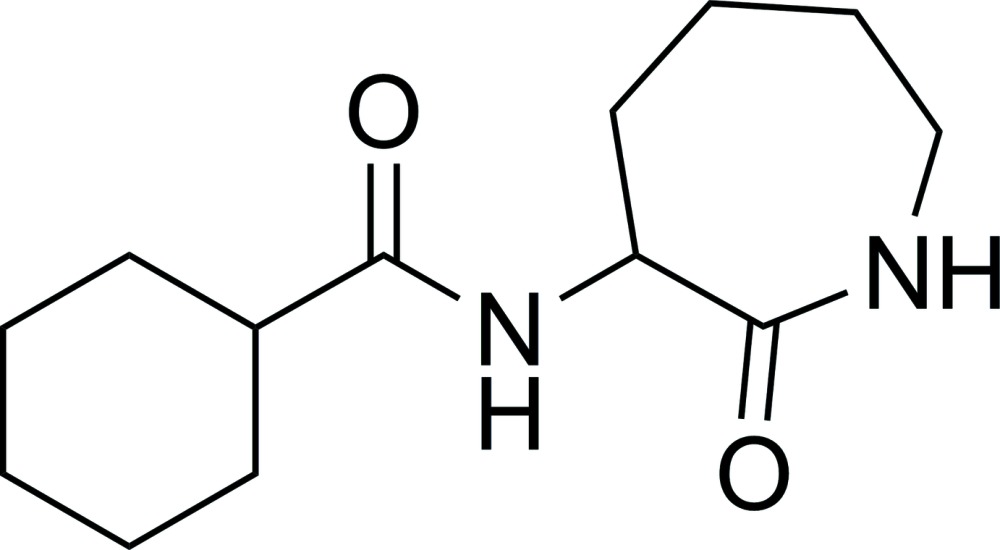


## Experimental

### 

#### Crystal data

C_13_H_22_N_2_O_2_
*M* *_r_* = 238.33
Triclinic, 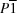

*a* = 5.007 (1) Å
*b* = 11.642 (2) Å
*c* = 12.739 (3) Å
α = 63.66 (3)°
β = 82.69 (3)°
γ = 82.75 (3)°
*V* = 658.0 (2) Å^3^
*Z* = 2
Mo *K*α radiation
μ = 0.08 mm^−1^
*T* = 293 K
0.30 × 0.20 × 0.10 mm

#### Data collection

Enraf–Nonius CAD-4 diffractometer
Absorption correction: ψ scan (North *et al.*, 1968 ▶) *T*_min_ = 0.976, *T*_max_ = 0.992
2700 measured reflections
2400 independent reflections
1581 reflections with *I* > 2σ(*I*)
*R*_int_ = 0.073
3 standard reflections every 200 reflections intensity decay: 1%

#### Refinement

*R*[*F* ^2^ > 2σ(*F* ^2^)] = 0.062
*wR*(*F* ^2^) = 0.177
*S* = 1.01
2400 reflections
154 parameters
H-atom parameters constrained
Δρ_max_ = 0.16 e Å^−3^
Δρ_min_ = −0.21 e Å^−3^

### 

Data collection: *CAD-4 EXPRESS* (Enraf–Nonius, 1989 ▶); cell refinement: *CAD-4 EXPRESS*; data reduction: *XCAD4* (Harms & Wocadlo, 1995 ▶); program(s) used to solve structure: *SHELXS97* (Sheldrick, 2008 ▶); program(s) used to refine structure: *SHELXL97* (Sheldrick, 2008 ▶); molecular graphics: *SHELXTL* (Sheldrick, 2008 ▶); software used to prepare material for publication: *PLATON* (Spek, 2009 ▶).

## Supplementary Material

Crystal structure: contains datablock(s) Y, I. DOI: 10.1107/S1600536813031863/pk2501sup1.cif

Structure factors: contains datablock(s) I. DOI: 10.1107/S1600536813031863/pk2501Isup2.hkl

Supplementary material file. DOI: 10.1107/S1600536813031863/pk2501Isup3.cml

Additional supplementary materials:  crystallographic information; 3D view; checkCIF report

## Figures and Tables

**Table 1:** Hydrogen-bond geometry (Å, °)

*D*—H⋯*A*	*D*—H	H⋯*A*	*D*⋯*A*	*D*—H⋯*A*
N1—H1*A*⋯O1^i^	0.86	2.37	3.158 (3)	152
N2—H2*A*⋯O2^ii^	0.86	2.09	2.927 (3)	165

Symmetry codes: (i) 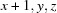; (ii) 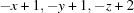.

**Fig. 1.**
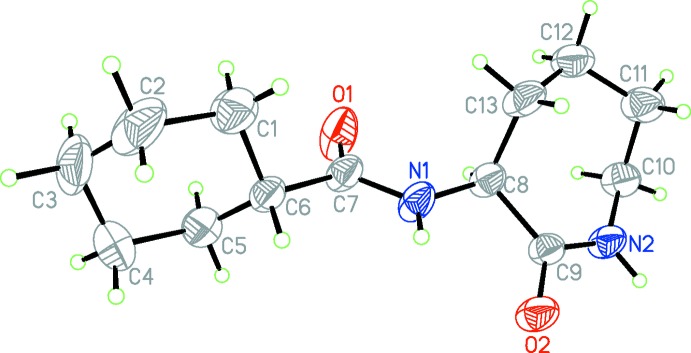
The molecular structure of the title compound. Displacement ellipsoids are drawn at the 50% probability level. H atoms are presented as small spheres of arbitrary radius.

**Fig. 2.**
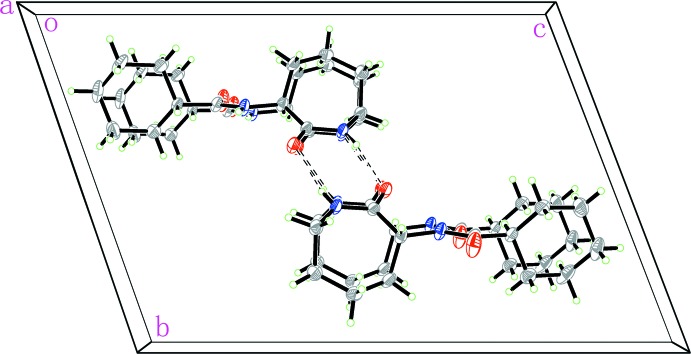
A view of the unit cell packing of the title compound, projected down the a axis. Hydrogen bonds are drawn as dashed lines.

## supplementary crystallographic information

### 1. Comment

*N*-(hexahydro-2-oxo-1*H*-azepin-3-yl)-cyclohexanecarboxamide (I), Fig. 1, is a 3-(acylamino)azepan-2-one, a series of compounds that are broad spectrum chemokine inhibitors and act as stable, orally available powerful anti-inflammatory agents (Fox *et al.*, 2009; Grainger *et al.*, 2006). We report herein the synthesis and crystal structure of the title compound (I).

In the crystal structure, molecules are linked by pairs of N—H···O hydrogen bonds between inversion-related lactam rings into centrosymmetric dimers with an R_2_^2^(8) graph-set motif (see Fig. 2).

### 2. Experimental

3-amino-caprolactam hydro-pyrrolidine-5-carboxylate (5 mmol) and Na_2_CO_3_ (15 mmol) in water (25 ml) were added to a solution of cyclohexanecarbonyl chloride (5 mmol) in CH_2_Cl_2_ (25 ml) at room temperature and the reaction was stirred for 12 h. The organic layer was then separated and the aqueous phase was extracted with additional CH_2_Cl_2_ (2 × 25 ml). The combined organic layers were dried over Na_2_CO_3_ and reduced *in vacuo*. The residue was purified by recrystallization from EtOAc / hexane to give the title compound (540 mg, 45% yield). (Grainger *et al.*, 2006).

Crystals of (I) suitable for X-ray diffraction were obtained by slow evaporation of an ethanol solution.

### 3. Refinement

All H atoms were positioned geometrically and refined using a riding model, with C—H = 0.98 and 0.97 Å, for methine and methylene H-atoms, and N—H = 0.86 Å, for amido H-atoms, respectively. The *U*_iso_(H) were allowed at 1.2*U*_eq_(C).

### Crystal data

**Table:**

C_13_H_22_N_2_O_2_	*Z* = 2
*M**_r_* = 238.33	*F*(000) = 260
Triclinic, *P*1	*D*_x_ = 1.203 Mg m^−^^3^
Hall symbol: -P 1	Mo *K*α radiation, λ = 0.71073 Å
*a* = 5.007 (1) Å	Cell parameters from 25 reflections
*b* = 11.642 (2) Å	θ = 9–13°
*c* = 12.739 (3) Å	µ = 0.08 mm^−^^1^
α = 63.66 (3)°	*T* = 293 K
β = 82.69 (3)°	Block, colorless
γ = 82.75 (3)°	0.30 × 0.20 × 0.10 mm
*V* = 658.0 (2) Å^3^	

### Data collection

**Table:**

Enraf–Nonius CAD-4 diffractometer	1581 reflections with *I* > 2σ(*I*)
Radiation source: fine-focus sealed tube	*R*_int_ = 0.073
Graphite monochromator	θ_max_ = 25.4°, θ_min_ = 1.8°
ω/2θ scans	*h* = 0→6
Absorption correction: ψ scan (North *et al.*, 1968)	*k* = −13→14
*T*_min_ = 0.976, *T*_max_ = 0.992	*l* = −15→15
2700 measured reflections	3 standard reflections every 200 reflections
2400 independent reflections	intensity decay: 1%

### Refinement

**Table:**

Refinement on *F*^2^	Primary atom site location: structure-invariant direct methods
Least-squares matrix: full	Secondary atom site location: difference Fourier map
*R*[*F*^2^ > 2σ(*F*^2^)] = 0.062	Hydrogen site location: inferred from neighbouring sites
*wR*(*F*^2^) = 0.177	H-atom parameters constrained
*S* = 1.01	*w* = 1/[σ^2^(*F*_o_^2^) + (0.095*P*)^2^] where *P* = (*F*_o_^2^ + 2*F*_c_^2^)/3
2400 reflections	(Δ/σ)_max_ < 0.001
154 parameters	Δρ_max_ = 0.16 e Å^−^^3^
0 restraints	Δρ_min_ = −0.21 e Å^−^^3^

### Special details

**Table:**

Geometry. All e.s.d.'s (except the e.s.d. in the dihedral angle between two l.s. planes) are estimated using the full covariance matrix. The cell e.s.d.'s are taken into account individually in the estimation of e.s.d.'s in distances, angles and torsion angles; correlations between e.s.d.'s in cell parameters are only used when they are defined by crystal symmetry. An approximate (isotropic) treatment of cell e.s.d.'s is used for estimating e.s.d.'s involving l.s. planes.
Refinement. Refinement of *F*^2^ against ALL reflections. The weighted *R*-factor *wR* and goodness of fit *S* are based on *F*^2^, conventional *R*-factors *R* are based on *F*, with *F* set to zero for negative *F*^2^. The threshold expression of *F*^2^ > σ(*F*^2^) is used only for calculating *R*-factors(gt) *etc*. and is not relevant to the choice of reflections for refinement. *R*-factors based on *F*^2^ are statistically about twice as large as those based on *F*, and *R*- factors based on ALL data will be even larger.

### Fractional atomic coordinates and isotropic or equivalent isotropic displacement parameters (Å^2^)

**Table:**

	*x*	*y*	*z*	*U*_iso_*/*U*_eq_	
N1	0.3592 (4)	0.2916 (2)	0.81058 (18)	0.0438 (6)	
H1A	0.5252	0.3096	0.7936	0.053*	
O1	−0.0132 (4)	0.2579 (2)	0.74942 (18)	0.0665 (7)	
C1	0.4450 (6)	0.1452 (3)	0.6317 (3)	0.0548 (8)	
H1B	0.5415	0.0899	0.7003	0.066*	
H1C	0.2722	0.1110	0.6407	0.066*	
O2	0.5524 (4)	0.42401 (18)	0.90229 (15)	0.0477 (5)	
N2	0.2504 (4)	0.3723 (2)	1.05959 (17)	0.0430 (6)	
H2A	0.3272	0.4210	1.0794	0.052*	
C2	0.6071 (7)	0.1457 (3)	0.5219 (3)	0.0714 (10)	
H2B	0.7850	0.1738	0.5161	0.086*	
H2C	0.6295	0.0592	0.5276	0.086*	
C3	0.4643 (7)	0.2349 (4)	0.4128 (3)	0.0727 (11)	
H3A	0.5718	0.2357	0.3436	0.087*	
H3B	0.2912	0.2037	0.4163	0.087*	
C4	0.4213 (8)	0.3700 (3)	0.4037 (3)	0.0719 (10)	
H4A	0.5951	0.4040	0.3930	0.086*	
H4B	0.3228	0.4249	0.3357	0.086*	
C5	0.2662 (6)	0.3715 (3)	0.5124 (2)	0.0508 (7)	
H5A	0.0850	0.3471	0.5176	0.061*	
H5B	0.2512	0.4582	0.5060	0.061*	
C6	0.3986 (5)	0.2813 (2)	0.6235 (2)	0.0376 (6)	
H6A	0.5742	0.3117	0.6210	0.045*	
C7	0.2294 (5)	0.2772 (2)	0.7325 (2)	0.0408 (6)	
C8	0.2270 (5)	0.2776 (2)	0.9237 (2)	0.0382 (6)	
H8A	0.0359	0.3077	0.9136	0.046*	
C9	0.3562 (5)	0.3637 (2)	0.9613 (2)	0.0353 (6)	
C10	0.0196 (5)	0.3088 (3)	1.1375 (2)	0.0468 (7)	
H10A	−0.1360	0.3313	1.0919	0.056*	
H10B	−0.0225	0.3411	1.1964	0.056*	
C11	0.0673 (7)	0.1631 (3)	1.1989 (2)	0.0549 (8)	
H11A	0.2469	0.1405	1.2267	0.066*	
H11B	−0.0617	0.1301	1.2670	0.066*	
C12	0.0410 (6)	0.0989 (3)	1.1204 (2)	0.0534 (8)	
H12A	−0.1396	0.1209	1.0937	0.064*	
H12B	0.0611	0.0064	1.1667	0.064*	
C13	0.2449 (6)	0.1356 (3)	1.0132 (2)	0.0479 (7)	
H13A	0.2224	0.0839	0.9731	0.057*	
H13B	0.4250	0.1130	1.0404	0.057*	

### Atomic displacement parameters (Å^2^)

**Table:**

	*U*^11^	*U*^22^	*U*^33^	*U*^12^	*U*^13^	*U*^23^
N1	0.0397 (12)	0.0635 (15)	0.0449 (12)	−0.0215 (10)	0.0136 (10)	−0.0387 (11)
O1	0.0423 (12)	0.1187 (19)	0.0656 (14)	−0.0274 (11)	0.0159 (10)	−0.0641 (14)
C1	0.0622 (18)	0.0482 (17)	0.0599 (18)	−0.0033 (14)	0.0042 (15)	−0.0314 (15)
O2	0.0554 (11)	0.0538 (11)	0.0469 (10)	−0.0262 (9)	0.0179 (9)	−0.0336 (9)
N2	0.0517 (13)	0.0472 (13)	0.0420 (12)	−0.0192 (10)	0.0138 (10)	−0.0306 (10)
C2	0.068 (2)	0.079 (2)	0.099 (3)	−0.0141 (18)	0.022 (2)	−0.071 (2)
C3	0.073 (2)	0.111 (3)	0.062 (2)	−0.033 (2)	0.0272 (18)	−0.065 (2)
C4	0.094 (3)	0.077 (2)	0.0431 (17)	−0.029 (2)	0.0159 (17)	−0.0247 (16)
C5	0.0596 (18)	0.0468 (16)	0.0490 (16)	−0.0079 (13)	0.0016 (14)	−0.0242 (13)
C6	0.0378 (13)	0.0428 (14)	0.0415 (14)	−0.0134 (11)	0.0080 (11)	−0.0270 (12)
C7	0.0373 (14)	0.0506 (16)	0.0474 (15)	−0.0141 (11)	0.0088 (12)	−0.0332 (13)
C8	0.0371 (13)	0.0455 (15)	0.0413 (14)	−0.0134 (11)	0.0101 (11)	−0.0281 (12)
C9	0.0370 (13)	0.0357 (13)	0.0369 (13)	−0.0098 (11)	0.0065 (11)	−0.0199 (11)
C10	0.0506 (16)	0.0488 (16)	0.0448 (15)	−0.0134 (12)	0.0154 (13)	−0.0263 (13)
C11	0.0694 (19)	0.0503 (17)	0.0468 (16)	−0.0218 (14)	0.0153 (15)	−0.0236 (14)
C12	0.0667 (18)	0.0439 (16)	0.0517 (17)	−0.0234 (14)	0.0096 (15)	−0.0215 (13)
C13	0.0565 (16)	0.0448 (16)	0.0565 (17)	−0.0137 (13)	0.0070 (14)	−0.0351 (14)

### Geometric parameters (Å, º)

**Table:**

N1—C7	1.334 (3)	C4—H4B	0.9700
N1—C8	1.455 (3)	C5—C6	1.513 (4)
N1—H1A	0.8600	C5—H5A	0.9700
O1—C7	1.238 (3)	C5—H5B	0.9700
C1—C2	1.523 (4)	C6—C7	1.516 (3)
C1—C6	1.530 (3)	C6—H6A	0.9800
C1—H1B	0.9700	C8—C9	1.524 (3)
C1—H1C	0.9700	C8—C13	1.536 (4)
O2—C9	1.241 (3)	C8—H8A	0.9800
N2—C9	1.337 (3)	C10—C11	1.522 (4)
N2—C10	1.462 (3)	C10—H10A	0.9700
N2—H2A	0.8600	C10—H10B	0.9700
C2—C3	1.519 (5)	C11—C12	1.516 (4)
C2—H2B	0.9700	C11—H11A	0.9700
C2—H2C	0.9700	C11—H11B	0.9700
C3—C4	1.515 (5)	C12—C13	1.526 (4)
C3—H3A	0.9700	C12—H12A	0.9700
C3—H3B	0.9700	C12—H12B	0.9700
C4—C5	1.505 (4)	C13—H13A	0.9700
C4—H4A	0.9700	C13—H13B	0.9700
			
C7—N1—C8	121.7 (2)	C5—C6—H6A	108.5
C7—N1—H1A	119.2	C7—C6—H6A	108.5
C8—N1—H1A	119.2	C1—C6—H6A	108.5
C2—C1—C6	110.7 (2)	O1—C7—N1	122.0 (2)
C2—C1—H1B	109.5	O1—C7—C6	121.9 (2)
C6—C1—H1B	109.5	N1—C7—C6	116.1 (2)
C2—C1—H1C	109.5	N1—C8—C9	108.04 (19)
C6—C1—H1C	109.5	N1—C8—C13	110.16 (19)
H1B—C1—H1C	108.1	C9—C8—C13	113.2 (2)
C9—N2—C10	127.8 (2)	N1—C8—H8A	108.5
C9—N2—H2A	116.1	C9—C8—H8A	108.5
C10—N2—H2A	116.1	C13—C8—H8A	108.5
C3—C2—C1	110.5 (3)	O2—C9—N2	121.3 (2)
C3—C2—H2B	109.5	O2—C9—C8	121.0 (2)
C1—C2—H2B	109.5	N2—C9—C8	117.7 (2)
C3—C2—H2C	109.5	N2—C10—C11	113.6 (2)
C1—C2—H2C	109.5	N2—C10—H10A	108.8
H2B—C2—H2C	108.1	C11—C10—H10A	108.8
C4—C3—C2	110.5 (3)	N2—C10—H10B	108.8
C4—C3—H3A	109.6	C11—C10—H10B	108.8
C2—C3—H3A	109.6	H10A—C10—H10B	107.7
C4—C3—H3B	109.6	C12—C11—C10	113.2 (2)
C2—C3—H3B	109.6	C12—C11—H11A	108.9
H3A—C3—H3B	108.1	C10—C11—H11A	108.9
C5—C4—C3	111.0 (2)	C12—C11—H11B	108.9
C5—C4—H4A	109.4	C10—C11—H11B	108.9
C3—C4—H4A	109.4	H11A—C11—H11B	107.8
C5—C4—H4B	109.4	C11—C12—C13	114.7 (2)
C3—C4—H4B	109.4	C11—C12—H12A	108.6
H4A—C4—H4B	108.0	C13—C12—H12A	108.6
C4—C5—C6	112.5 (2)	C11—C12—H12B	108.6
C4—C5—H5A	109.1	C13—C12—H12B	108.6
C6—C5—H5A	109.1	H12A—C12—H12B	107.6
C4—C5—H5B	109.1	C12—C13—C8	116.1 (2)
C6—C5—H5B	109.1	C12—C13—H13A	108.3
H5A—C5—H5B	107.8	C8—C13—H13A	108.3
C5—C6—C7	111.7 (2)	C12—C13—H13B	108.3
C5—C6—C1	110.5 (2)	C8—C13—H13B	108.3
C7—C6—C1	109.0 (2)	H13A—C13—H13B	107.4
			
C6—C1—C2—C3	−57.4 (3)	C7—N1—C8—C9	−150.4 (2)
C1—C2—C3—C4	57.9 (3)	C7—N1—C8—C13	85.5 (3)
C2—C3—C4—C5	−56.5 (4)	C10—N2—C9—O2	179.3 (2)
C3—C4—C5—C6	55.4 (4)	C10—N2—C9—C8	−0.4 (4)
C4—C5—C6—C7	−176.0 (2)	N1—C8—C9—O2	−4.5 (3)
C4—C5—C6—C1	−54.5 (3)	C13—C8—C9—O2	117.8 (3)
C2—C1—C6—C5	55.1 (3)	N1—C8—C9—N2	175.3 (2)
C2—C1—C6—C7	178.2 (2)	C13—C8—C9—N2	−62.5 (3)
C8—N1—C7—O1	3.9 (4)	C9—N2—C10—C11	65.1 (3)
C8—N1—C7—C6	−174.2 (2)	N2—C10—C11—C12	−78.0 (3)
C5—C6—C7—O1	51.0 (3)	C10—C11—C12—C13	62.3 (3)
C1—C6—C7—O1	−71.4 (3)	C11—C12—C13—C8	−63.3 (3)
C5—C6—C7—N1	−131.0 (3)	N1—C8—C13—C12	−160.1 (2)
C1—C6—C7—N1	106.7 (3)	C9—C8—C13—C12	78.9 (3)

### Hydrogen-bond geometry (Å, º)

**Table:**

*D*—H···*A*	*D*—H	H···*A*	*D*···*A*	*D*—H···*A*
N1—H1*A*···O1^i^	0.86	2.37	3.158 (3)	152
N2—H2*A*···O2^ii^	0.86	2.09	2.927 (3)	165

Symmetry codes: (i) *x*+1, *y*, *z*; (ii) −*x*+1, −*y*+1, −*z*+2.

## References

[bb1] Enraf–Nonius (1989). *CAD-4 Software* Enraf–Nonius, Delft, The Netherlands.

[bb2] Fox, D. J., Reckless, J., Lingard, H., Warren, S. & Grainger, D. J. (2009). *J. Med. Chem.* **52**, 3591–3595. 10.1021/jm900133w 19425597

[bb3] Grainger, D. J. & Fox, D. J. (2006). Patent WO 2006016152A1.

[bb4] Harms, K. & Wocadlo, S. (1995). *XCAD4* University of Marburg, Germany.

[bb5] North, A. C. T., Phillips, D. C. & Mathews, F. S. (1968). *Acta Cryst.* A**24**, 351–359.

[bb6] Sheldrick, G. M. (2008). *Acta Cryst.* A**64**, 112–122. 10.1107/S0108767307043930 18156677

[bb7] Spek, A. L. (2009). *Acta Cryst.* D**65**, 148–155. 10.1107/S090744490804362X PMC2631630 19171970

[bb8] Zhu, N., Tran, P., Bell, N. & Stevens, C. L. K. (2007). *J. Chem. Crystallogr.* **37**, 670–683.

